# Astragalin: a food-origin flavonoid with therapeutic effect for multiple diseases

**DOI:** 10.3389/fphar.2023.1265960

**Published:** 2023-10-18

**Authors:** Junren Chen, Kexin Zhong, Siqi Qin, Yiqi Jing, Shengmeng Liu, Dan Li, Cheng Peng

**Affiliations:** State Key Laboratory of Southwestern Chinese Medicine Resources, School of Pharmacy, Chengdu University of Traditional Chinese Medicine, Chengdu, China

**Keywords:** Astragalin, pharmacological activities, diseases, molecular mechanisms, natural source

## Abstract

Naturally occurring flavonoids have long been utilized as essential templates for the development of novel drugs and as critical ingredients for functional foods. Astragalin (AG) is a natural flavonoid that can be isolated from a variety of familiar edible plants, such as the seeds of green tea, Morus alba L., and Cuscuta chinensis. It is noteworthy that AG has a wide range of pharmacological activities and possesses therapeutic effects against a variety of diseases, covering cancers, osteoarthritis, osteoporosis, ulcerative colitis, mastitis, obesity, diabetes mellitus, diabetic complications, ischemia/reperfusion injury, neuropathy, respiratory diseases, and reproductive system diseases. This article reviewed the natural source and pharmacokinetics of AG and systematically summarized the pharmacological activities and potential mechanisms of AG in treating diverse diseases in order to promote the development of AG as a functional food, in doing so providing references for its clinical application in disease therapy.

## 1 Introduction

Natural products represent the chemical substances that are produced in nature by living organisms with specific pharmacological or biological activity, among which flavonoids occupy an essential position and are widely used in a wide range of foods or drugs as paramount ingredients (Shen et al., 2022). Astragalin (AG), also known as kaempferol 3-O-β-D-glucopyranoside (C_21_H_20_O_11_), with the molecular weight of 448.38, the melting point of 223°C–229 °C, and the boiling point of 823.2°C ± 65.0°C, is a pivotal flavonoid derived from the leaves of persimmon (Harikrishnan et al., 2020), the seeds of green tea, horseradish tree leaves (Zilla et al., 2014), lotus leaf (Oldoni et al., 2019), Chinese rose (Li et al., 2020), *Cuscuta chinensis* (Donnapee et al., 2014), *Morus alba L.*, and *Thesium chinense* (Tian et al., 2022). Some of the primary natural sources of AG are shown in Table 1. AG has emerged as a research hotspot in recent years due to its outstanding bioactivities and promising therapeutic effects for numerous diseases. So far, various pharmacologic abilities have been found, including anti-inflammatory effects (Soromou et al., 2012; Li et al., 2014; Zhang et al., 2017; Alblihed, 2020; Hu et al., 2020), anti-oxidative effects (Choi et al., 2013; Li, Tian, et al., 2017; Alblihed, 2020), procoagulant effects (Li et al., 2020), antibacterial effects (Ivanov et al., 2020), and analgesic effects (Li et al., 2017; Wang et al., 2020).

**TABLE 1:** The natural sources of astragalin.

*Species*	*Familla*	Part used	Reference
persimmon	Ebenaceae	leaves	Han et al. (2021)
			Zhao et al. (2021)
			Liu et al. (2020)
			Kim et al. (2017)
Rosa	*Rosaceae*	leaves	Han et al. (2021)
			Ma et al. (2015)
			Liu et al. (2020)
*Moringa oleifera*	*Moringaceae*	leaves	Peng et al. (2020)
			Muni Swamy et al. (2022)
*Astragalus membranaceus*	*Fabaceae*	root	Peng et al. (2020)
			Ke et al. (2012)
*Morus alba*	*Moraceae*	fruit	Peng et al. (2020)
			Wei et al. (2016)
*Cassia alata*	*Fabaceae*	leaves	Peng et al. (2020)
Gynura procumbens	*Asteraceae*	leaves	Rey et al. (2019)
Camellia sinensis	*Theaceae*	seeds	Qu et al. (2016)
			Kim et al. (2017)
*Cuscuta chinensis*	*Convolvulaceae*	seeds	Han et al. (2019)
			Fan et al. (2022)
			Karna et al. (2019)
			Meng et al. (2019)
*Prunus serotina*	*Rosaceae*	fruit	Hu et al. (2022)
*Flaveria bidentis*	*Asteraceae*	aerial part	Shaheen et al. (2015)

**FIGURE 1:**
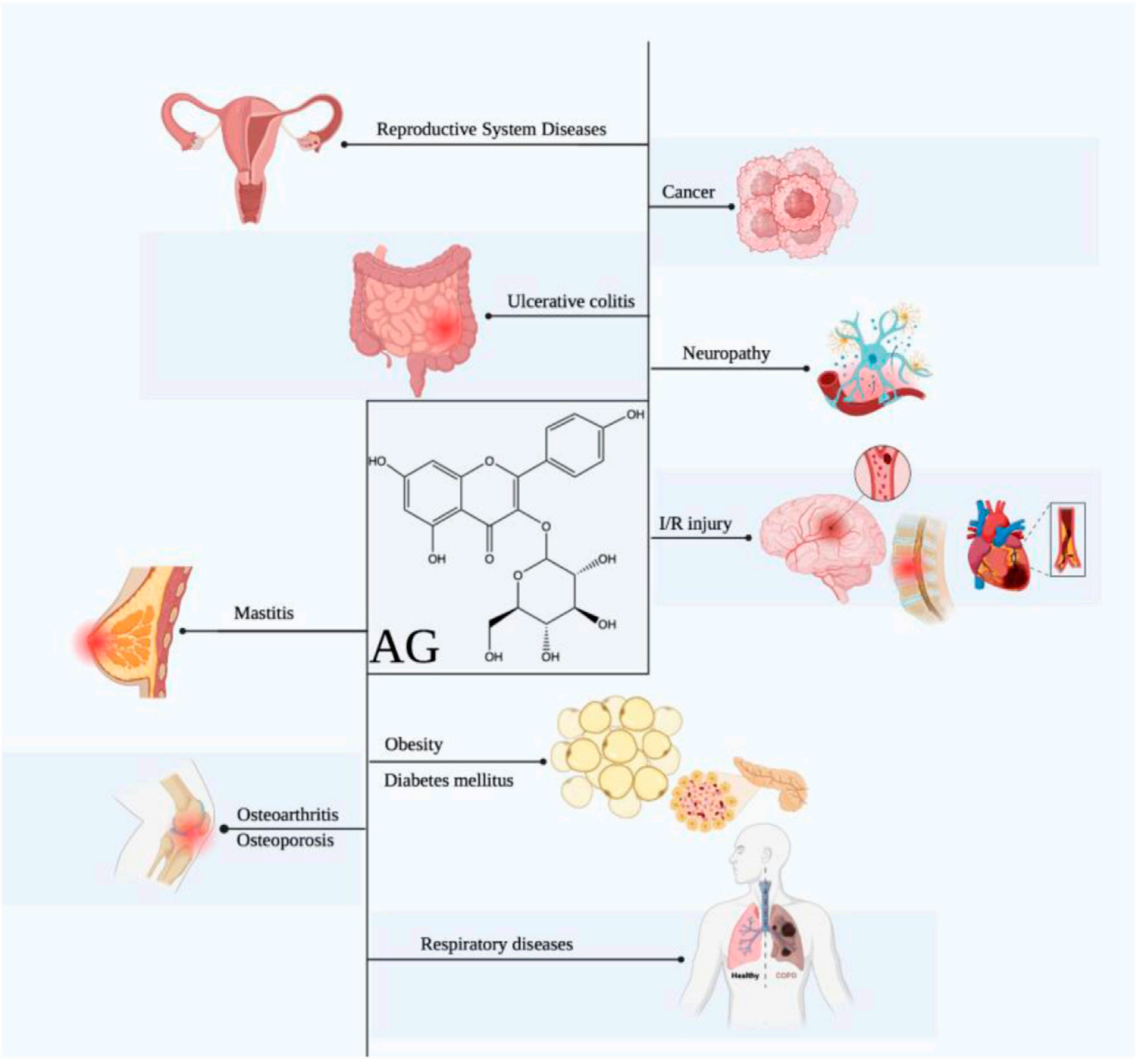
Therapeutic effects of AG against multiple diseases. (AG displays promising effects in treating diverse diseases, including cancers, obesity, DM, diabetic complications, ischemia/reperfusion injury, neuropathy, respiratory diseases, osteoarthritis, osteoporosis, ulcerative colitis, mastitis, and reproductive system diseases.)

Based on these pharmacological abilities, considerable studies focus on the potential of AG in diverse diseases, including cancers, obesity, diabetes mellitus (DM), diabetic complications, ischemia/reperfusion injury, neuropathy, respiratory diseases, osteoarthritis, osteoporosis, ulcerative colitis, and others (Figure 1). Furthermore, some molecular mechanisms of AG for the above diseases have been clarified, including suppressing inflammation and oxidative stress *via* targeting the TLR4/NF-κB pathway, alleviating pain *via* modulating the ERK pathway, inhibiting tumors *via* functioning the PI3K/AKT, MAPK, and JAK/STAT pathways, ameliorating neuropathy *via* modulating the HO-1/MAPK, P13K/Akt, SIRT1, and Notch/HES-1-NF-κB pathways, attenuating respiratory diseases *via* targeting the TLR4-PKCβ2-NADPH and MAPK pathways, improving osteoarthritis and osteoporosis *via* mediating the BMP pathway, and treating ulcerative colitis *via* modulating the NF-κB pathway (Table 2).

**TABLE 2:** Potential molecular mechanisms of AG in treating various diseases.

Cancers				
**Cancer in digestive system**				
Cells/models	Administration route	Dosages	Mechanisms (index)	References
**HCT116**	-	5, 10, 20, 40, 80, and 160μM	↑: caspase-3, caspase-6, caspase-7, caspase-8, caspase-9, P53, Bax, P21, P27 ↓: MMP-2, MMP-9, cleaved caspase-3, Bcl-2, CDK2, CDK4, Cyclin D1, Cyclin E, p-NF-κB, NF-κBP65, p-IκBα, TNF-α, IL-6, p-iκκα	Yang et al. (2021)
**BALB/c nude mice with HCT116 cells**	i.g	25, 50, and 75 mg/kg		
**AGS**	-	80 and 160μM	↓: MUC1, ppGalNAcT2, Tn, ST6GalNAcT2, sialyl Tn, C1GalT1, T, St3Gal-IV, FUT4, NF-κB	Radziejewska et al. (2022)
**gastric cancer cell lines**	-	10, 20, 40, and 80 μM	↑: PARP, Bim ↓: Bcl-2, p-PI3K, p- AKT	(Wang et al., 2021)
**nude mice with gastric cancer cell**	i.g	25 and 50 mg/kg		
skin cancer				
Cells/models	Administration route	Dosages	Mechanisms (index)	References
**A375P and SKMEL-2 cells**	-	12.5, 25, 50, 75, and 100 μM	↑: sub-G1 ↓: procaspase 9, cyclin D1, Mcl-1, SOX10	You et al. (2017)
Breast cancer				
Cells/models	Administration route	Dosages	Mechanisms (index)	References
**BC cells**	-	1, 10, 20, and 50 μM	↓: VEGFR, COX-2, ZEB1, p-AKT, MMP9	Tian et al. (2022)
Lung cancer				
Cells/models	Administration route	Dosages	Mechanisms (index)	References
**A549 and H1299**	-	5, 10, 20, and 40μM	↑: caspase-8, caspase-9, caspase-3, PARP, Bad, Bax, p-JNK ↓: Bcl-xl, Bcl-2, p-p38, p-ERK, p-PI3K, p-Akt, IκBα, p-IKK-β, Fas	Chen et al. (2017)
**nude mice with lung cancer cell**	i.g	20 and 50 mg/kg		
**NSCLC cells**	-	5, 25, 75, and 150 µM	↑: caspases-3, caspases-9, Bax, Bak, Cyt-C, ROS ↓: Bcl-2, Bcl-xL, XIAP, p-JAK2, p-STAT1, p-STAT3	Xu et al. (2021)
Liver cancer				
Cells/models	Administration route	Dosages	Mechanisms (index)	References
**HCC cells**	-	11 and 33μM	↑: Sub-G1, ROS, MDA, miR-125b ↓: HK2	Li et al. (2017)
**nude mice with liver cancer cell and Kunming mice transplanted with HCC cells**	i.g	10 and 20 mg/kg		
Kidney cancer				
Cells/models	Administration route	Dosages	Mechanisms (index)	References
**kidney cancer cell line**s	-	3.12, 6.25, 12.5, 25, 50, 100, and 200μM	↑: Caspase-3, Caspase-9, Bax, microRNA-124, microRNA-203, microRNA-34a, microRNA-145 ↓: Bcl-2	Zhu et al. (2019)
Obesity, diabetes mellitus, and diabetic complications				
Cells/models	Administration route	Dosages	Mechanisms (index)	References
**3T3-L1**	-	5, 10, 15, and 20 mg/mL	↑: adiponectin ↓: PPAR-γ, C/EBP-α, FAS, leptin	Muni Swamy et al. (2022)
**HG induced in pancreatic islets form diabetic rats**	-	25, 50, 100, and 150 μM	↑: Ca^2+^, PKC, PKA	Rey et al. (2019)
**HG-induced diabetic rats**	i.g	1 and 10 mg/kg		
**HG induced in Müller cells**	-	400 mg/L	↓: VEGF	Ke et al. (2012)
Obesity, diabetes mellitus, and diabetic complications				
Cells/models	Administration route	Dosages	Mechanisms (index)	References
**3T3-L1 cells**	-	10, 20, and 30 μM/mL	↓: PPARG, C/EBPα, FKBP51	Barge et al. (2021)
**HFD-induced rats**	i.g	200 mg/kg		
I/R injury				
Cells/models	Administration route	Dosages	Mechanisms (index)	References
**Isolated hearts of rats**	immersion	5, 10, and 20 𝜇mol/L	↑: SOD, GSH/GSSG, Bcl-2, LVDP, HR, CF ↓: LDH, CK, MDA, ROS, TNF-α, IL-6, Bax, IS	Qu et al. (2016)
**TG-induced in SH-SY5Y**	-	10,25,50, and 100 µM	↑: Bcl-2 ↓: IL-1β, IL-6, IL-8, TNF-α, COX-2, NOS, Bax, c-caspase-3, GRP78, CHOP, caspase12	Liu et al. (2020)
**I/R injury rats**	i.g	50 mg/kg/day		
**SCI/R injury mice**	i.t	1 mg/kg	↑: SOD, GSH ↓: MDA, TNF-α, IL-6, HMGB1, RIP1, RIP3, MLKL	Sun et al. (2021)
Neuropathy				
Cells/models	Administration route	Dosages	Mechanisms (index)	References
**LPS + ATP induced in BV2 cells**	-	10, 20, and 40 μM	↑: SIRT1↑, ↓: NF-κB p65, NLRP3, IL-1β, gasdermin D, caspase-1, Iba1	Tong et al. (2020)
**CUMS-challenged mice**	-	5 and 10 mg/kg		
**AlCl**_**3**_ **/Gal-treated mice**	s.c	5, 10, and 20 mg/kg	↑: T-SOD, T-AOC, CAT, GSH-Px, IL-10, APP ↓: MDA, TNF-α, IL-1β, IL-6, β-GAL, Notch1, HES-1, RBP-Jκ, NF-κB	Hu et al. (2022)
**Aβ1−42O-induced neuronal cell**	-	12.5, 25, 50, 100, and 200 µM	↑: GSK3β, ERα, ERβ ↓: Aβ_1−40_, Aβ_1−42_, β-CTF, Thr231, Ser396, Ser202/Thr205	Liu et al. (2022)
**SAMP8 mice**	i.g	5 and 10 mg/		
**LPS-induced in microglia**	-	2.5–10.0 μg/mL	↑: HO-1 ↓: NO, iNOS, TNF-α, IL-1β, p-p38, p-Erk, p-JNK, ROS, IL-6, COX-2, p-NF-κB	Kim et al. (2022)
**LPS-induced mice**	i.p	5 and 20 mg/kg		
**HEK293 cells**	-	50 µM	↓: P2X4, GFAP, TNF-R1, pERK1/2	Wang et al. (2020)
**CCl rats**	i.g	25, 50, and 100 mg/kg		
Respiratory diseases				
Cells/models	Administration route	Dosages	Mechanisms (index)	References
**BEAS-2B cells**	-	1–20 μM	↓: MCP-1, ICAM-1, integrins, F4/80, CD68, CD11b, α-SMA	Kim et al. (2017)
**OVA-induced COPD mice**	i.g	10 and 20 mg/kg		
**A549 cells**	-	1–20 μM	↑: tPA ↓: PAR-1, PAR-2, uPA, TF, CD11b, F4/80, COX-2, iNOS, ROS, p-p38, p-c-Jun, p-JNK, p-ERK	Kim et al. (2021)
**Cigarette smoke-induced COPD mice**	i.g	10 and 20 mg/kg		
**BEAS-2B cells**	-	25, 50, 100, and 200μM	↑: Nrf2, HO-1 ↓: MPO, TNF-α, MMP-9	Zheng et al. (2019)
**LPS-induced ALI rats**	Intratracheal administration	50 mg/kg		
**OVA-induced asthma mice**	i.g	0.5 and 1 mg/kg	↑: IFN-γ, SOCS-5 ↓: IL-4, IL-5, IL-13, IgE, SOCS-3	Liu et al. (2015)
**BEAS-2B-cell**	-	1, 10, and 20 μM	↑: p-ERK, p-Akt ↓: TLR4, ROS, eotaxin-1, PLCγ1, p-PKCβ2, p22^phox^, p47^phox^, p-p38, p-JNK, caspase-3	Cho et al. (2014)
**BEAS-2B-cell**	-	1, 10, and 20 μM	↓: ROS, beclin-1, LC3A/B, α-SMA	Cho et al. (2015)
**OVA-induced mice**	i.g	10 and 20 mg/kg		
**Osteoarthritis and Osteoporosis**				
Cells/models	Administration route	Dosages	Mechanisms (index)	References
**IL-1β-induced in human osteoarthritis chondrocyte**	-	20, 40, and 80 μg/mL	↑: PPAR-γ ↓: NO, PGE2, iNOS, COX-2, p65, β-actin, ERK1/2, JNK, p38	Ma et al. (2015)
**ovariectomy (OVX)-induced osteoporosis in mice**	i.g	2 and 4 g/kg	↑: BMD, OPG ↓: TRACP-5b, RANKL, ACP5, F2R, NFATC1, CTSK, c-fos, c-Src kinase	Yang et al. (2022)
**MC3T3-E1 cells**	-	5, 10, and 20µM	↑: ALP, Alp, Ocn, Opn, BMP-2, p-Smad1/5/9, Runx2, Erk1/2, p-Erk1/2, p38, p-p38, JNK, BMD↑, BV/TV, Tb.Th., Tb.N	Liu et al. (2019)
**ovariectomized (OVX)-induced osteoporotic mice**	i.p	5, 10, and 20 mg/kg		
Ulcerative colitis				
Cells/models	Administration route	Dosages	Mechanisms (index)	References
**DSS-indued acute colitis mice**	i.g	50, 75, and 100 mg/kg	↑: ZO-1, Occludin, Muc2 ↓: MCP-1, TNF-α, IL-1β, IL-6, IFN-γ, COX-2, MPO, LPS, TLR4, p-IκBα, p-IKKα/β, p-p65	Peng et al. (2020)
**HCT-116 and HT-29 cells**	-	10 and 50 μM	↓: TNF-α, IL-6, IL-8, IκBα	Han et al. (2021)
**DSS-induced acute colitis mice**	i.g	2 and 5 mg/kg		
Reproductive System Diseases				
Cells/models	Administration route	Dosages	Mechanisms (index)	References
**ovarian granulosa cells**	-	0.05, 0.1, 0.25, and 0.5 mM	↑: E2, P4, Bcl-2, Bcl-2/Bax ↓: FSH, LH	Wei et al. (2016)
**Aged female rats**	i.g	7, 35, and 70 μM/kg		
**STZ-induced spermatogenic dysfunction rats**	i.g	3.3, 10, and 30 mg/kg	↑: SOD, GSH-Px, CAT ↓: HbA1c, MDA, NO	Han et al. (2019)
**CTX-induced mice**	i.g	30 mg/kg	↑: AKT1, BCL-2, ETV1, MAPKAPK2, RPS6KA5, STAR, PRKACB, CYP11A1 ↓: BAD, BCL2-XL, CASPASE9, CASPASE3	Fan et al. (2022)
**varicocele rats**	p.o	200 mg/kg	↑: SOD, GPx, StAR ↓: ROS/RNS, MDA, TNF-α, IL-6, LH, FSH, Grp 78, p-IRE1, P-JNK, caspase-3, Bax/Bcl2	Karna et al. (2019)
**CYP1B1 treated with E2**	-	0.005, 0.05, 0.5, 5, and 50μM		Meng et al. (2019)
Mastitis				
Cells/models	Administration route	Dosages	Mechanisms (index)	References
**LPS-induced mouse mastitis model**	i.p	10, 25, and 50 mg/kg	↓: MPO, TNF-α, IL-1β, IL-6, NF-κB p65, IκBα	Li et al. (2013)
Other Effects				
Cells/models	Administration route	Dosages	Mechanisms (index)	References
**HaCaT cell lines**	-	20, 50, 100, and 200 µM	↓: p-H2AX, p-MSK1, p-p38, γ-H2AX, TNF-α	Li et al. (2018)
**UVB-induced in mice**	topically applied	50 mg/kg		
**A blood sample form rabbit**	-	0.3125, 0.6250, 1.250, 2.500, and 5.000 mg/mL	↑: PLC, TXB_2_, ET-1, PV, PCV, ESR, WBV ↓: APTT, TT, FIB, CT, 6-keto-PGF_1α_, eNOS, PT	Li et al. (2020)
**heparin sodium-induced rats**	i.v	2.5, 5, and 10 mg/kg		
Other Effects				
Cells/models	Administration route	Dosages	Mechanisms (index)	References
***E. coli* induced in SEECs**	-	12.5, 25, 50, 100, 200, and 400µM	↓: IKKβ, NF-κB, IL-1β, IL-6, IL-8, IL-12α, TNF-α, TLR4, p-IκBα/IκBα	Hu et al. (2020)
**LPS-induced endotoxemia mice**	i.g	25, 50, and 75 mg/kg	↓: W/D ratio, TNF-α, IL-1β, IL-6, IκB	Soromou et al. (2012)
**LPS-induced ALI mice**	i.g	50 and 75 mg/kg		
**LPS-induced in uterine epithelium cells**	-	100μM	↓: TNF-α, IL-1β, IL-6, p-IκB, p-p65, p38, ERK, JNK	Zhang et al. (2017)
**Leptospira-induced uterine inflammation in mice**		50 mg/kg		
**Carrageenan-induced paw edema in mice**	i.g	75 mg/kg	↑: SOD, CAT ↓: MDA, iNOS, NO, COX-2, PGE2, MCP-1, MPO, TNF-α, IL-1β, IL-6, NF-κB	Alblihed (2020)
**MH7A cells**	-	50, 100, and 200 µM	↓: TNF-α, IL-1β, IL-6, IL-8, MMP-1, MMP-3, MMP-13, p-JNK, p-p38, p-c-Jun	Jia et al. (2019)
**Collagen-induced arthritis mice**	i.p	5 mg/kg/day		

Notably, different skeleton structures and the substitution of phenolic hydroxyl groups affect the anti-inflammatory effects of flavonoid glycosides (Wang et al., 2021). With the same phenolic hydroxyl substitutions, *in vitro* experiments demonstrated that both low concentrations of aglycone of AG (apigenin) and high concentrations of flavanone naringenin can produce the same anti-inflammatory effect, and the inhibitory effect of the flavonol Kaempferol on TNF-α was around 20% less than that of apigenin at the same concentration. With the same flavonoid structure, the flavonoids with 5,7-OH substitution on the A ring or only one hydroxyl substitution on the B ring exhibited superior anti-inflammatory effects. The inhibitory effect of one hydroxyl substitution (apigenin) on NO production was superior to that of zero hydroxyl substitution (chrysin) and two hydroxyl substitutions (luteolin). These findings illustrated that AG might possess superior anti-inflammatory effects to other flavonoids.

This article reviewed the biological activities and the capacities of AG in treating multiple diseases, aiming to provide mechanistic references for clinical studies of AG and to contribute to the development of AG as a novel drug or functional food.

## 2 Pharmacological effects of AG

### 2.1 Effect on cancers

**FIGURE 2:**
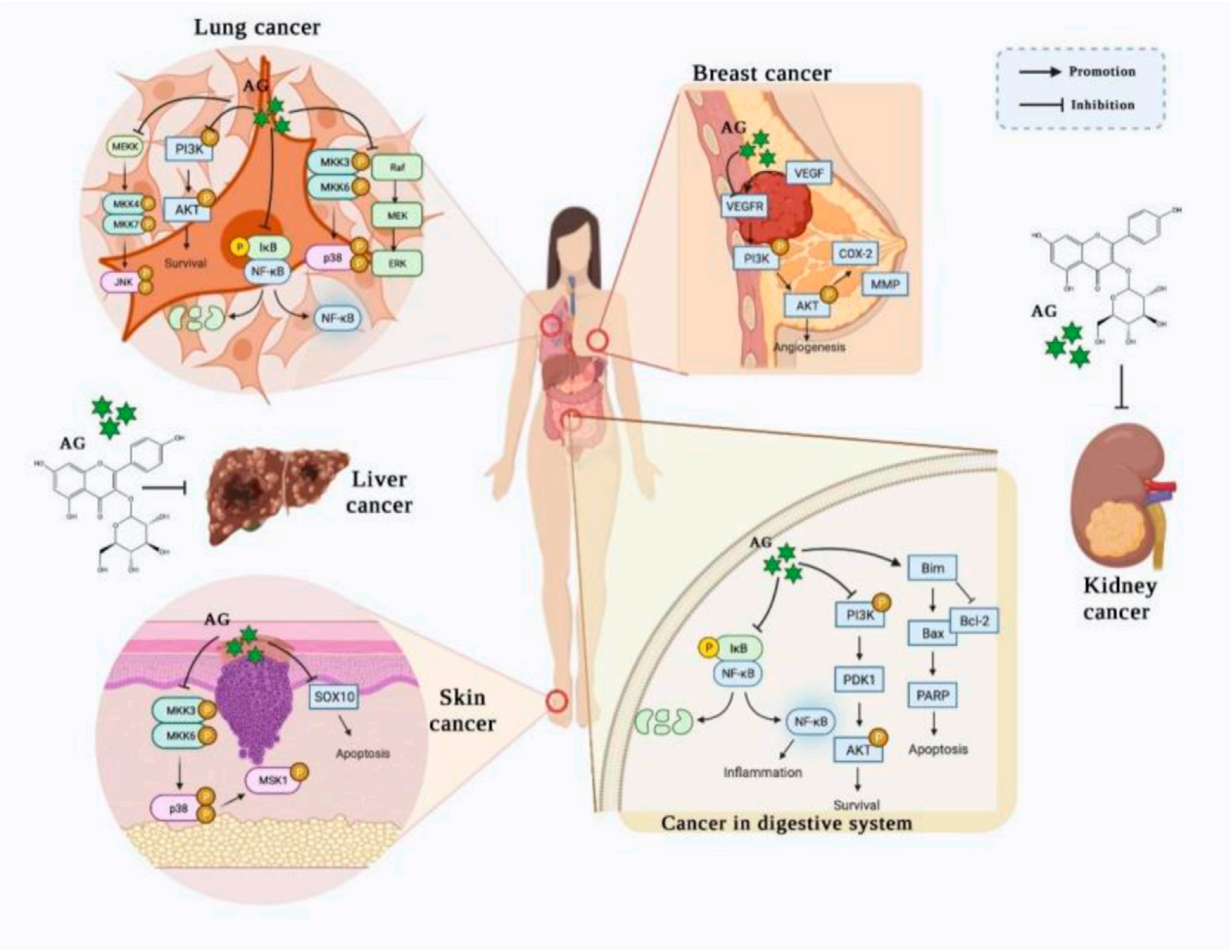
The potential effects of AG on various cancers. (AG treats/prevents lung cancer, breast cancer, skin cancer, gastric cancer, and colon cancer by promoting apoptosis and attenuating the growth of tumor cells, principally *via* mediating the NF-κB, PI3K/AKT, MAPK, and JAK/STAT signaling pathways.)

An increasing number of studies have demonstrated that AG could prevent numerous types of cancers, especially cancers of the digestive system, skin, breast, lung, liver, and kidney. Remarkably, these findings partly uncover the underlying mechanisms, including promoting the apoptosis and attenuating the growth of tumor cells *via* mediating the NF-κB, PI3K/AKT, MAPK, and JAK/STAT signaling pathways (Figure 2).

#### 2.1.1 Cancer in digestive system

AG has been shown to suppress gastric cancer cells growth, invasion, and migration by down-regulating gastric epithelium glycoprotein MUC1 and tumor-associated antigens. Specifically, Radziejewska *et al.* observed that AG blocked the synthesis of Tn antigen, sialyl Tn antigen, and T antigen *via* suppressing the expressions of the glycosyltransferases ppGalNAcT2, ST6GalNAcT2, C1GalT1, St3Gal-IV, and FUT4 in AGS cells. Moreover, AG also decreased the level of NF-κB, which suggests that the NF-κB signaling pathway might be involved in the inhibition of the glycosylation process (Radziejewska et al., 2022). In addition, AG was reported to induce the death of gastric cancer cells and reduce cancer cell viability in a gastric cancer cell-based xenograft mouse without obvious toxicity. AG administration promoted cell apoptosis and attenuated cell proliferation by up-regulating PARP and Bim and down-regulating Bcl-2, p-PI3K, and p-AKT (Wang et al., 2021), indicating that activating PI3K/AKT signaling is a promising mechanism of AG in preventing gastric cancer.

Moreover, a recent study proved that AG could prevent nude mice from colon cancer cells *via* inactivating the NF-κB pathway. AG intervention suppressed the volumes of colon tumor *in vivo* and downregulated the phosphorylation of IKKα and NF-κB. Furthermore, *in vitro* experiments revealed that AG restrained the growth and migration of HCT116 cells by down-regulating the expressions of MMP-2 and MMP-9 and suspended the cell cycle of G0/G1 phase *via* declining the levels of CDK2, CDK4, Cyclin D1, and Cyclin E and augmenting P21 and P27; meanwhile, it induced cell apoptosis by increasing the levels of caspase-3, caspase-6, caspase-7, caspase-8, caspase-9, P53, and Bax and decreased cleaved caspase-3 and Bcl-2. In addition, AG also downregulated p-NF-κB, NF-κBP65, p-IκBα, TNF-α, and IL-6 (Yang et al., 2021), indicating that targeting NF-κB signaling might be one of the mechanisms of AG in colon cancer therapy. Collectively, these studies elucidated that AG exhibits anti-tumor effects in the digestive system *via* modulating the NF-κB and PI3K/AKT signaling pathways.

#### 2.1.2 Skin cancer

AG has been shown to contribute to the death of melanoma skin cancers cells by promoting the apoptosis of cells and reducing cell viability. AG administration enhanced A375P and SKMEL-2 melanoma cells apoptosis by elevating the TUNEL-positive cells number, up-regulating the expression of sub-G1, increasing PARP, cleaved caspase-9, and caspase-3, and down-regulating procaspase 9, cyclin D1, Mcl-1, and SOX10. Notably, SOX10 overexpression blocked the pro-apoptotic effect of AG, suggesting that targeting SOX10 and apoptotic-related proteins may be the mechanism of AG in preventing melanoma skin cancer (You et al., 2017). Therefore, these results revealed that AG suppressed skin cancer by triggering cancer cell apoptosis and suppressing the p38 MAPK signaling pathway.

#### 2.1.3 Breast cancer

Breast cancer remains the most prevalent invasive malignancy and the second leading contributor to cancer mortality in women, despite multiple treatment strategies and options having been investigated (Britt, Cuzick, and Phillips, 2020; Jokar et al., 2021). Cytochrome P450 (CYP) 1B1 has been demonstrated to be involved in the metabolism of estradiol and to catalyze the formation of 4-hydroxy-E2 (4-OH-E2), and 4-OH-E2 exhibits a tumorigenic effect, which has been revealed to contribute to the occurrence and development of breast cancer (Wu et al., 2021). Increasing evidence has illustrated that inhibiting the formation of 4-OH-E2 by binding to a CYP1B1 activity site or the CYP1B1-catalyzed enzymes (Meng et al., 2019) might be a pivotal mechanism of AG in alleviating breast cancer. AG intervention has been shown to attenuate breast cancer cell metastasis and new blood vessel formation in HUVECs *via* decreasing the expressions of VEGFR, COX-2, ZEB1, p-AKT, and MMP9. Moreover, AG also blocks angiogenesis *in vivo* according to a CAM assay (Tian et al., 2022). Taken together, these findings suggest that AG prevents breast cancer by modulating estradiol metabolism and suppressing the expressions of MMP9 and proteins associated with angiogenesis.

#### 2.1.4 Lung cancer

AG also displays an outstanding therapeutic effect in lung cancer, which has been shown to facilitate lung cancer cell death and suppress the growth of cell *via* modulating the MAPK and NF-кB pathways (Li et al., 2023). Chen et al. reported that AG restrained the growth and weight of tumor in xenograft nude mice, elevated the number of apoptosis cells, and inhibited clonogenic cell growth. AG treatment increased the expressions of caspase-8, caspase-9, caspase-3, PARP, Bad, Bax, and Fas and decreased Bcl-xl and Bcl-2. Moreover, AG application inactivated the MAPK pathway and attenuated LPS, and TNFα induced NF-кB pathway activation *via* down-regulating p-p38, p-ERK, p-PI3K, p-Akt, IκBα, and p-IKK-β and up-regulating p-JNK in A549 cells. Interestingly, the inhibitor of MEK and PI3K/Akt mimicked the effects of AG, whereas the antagonist of caspase abolished the pro-apoptotic effect of AG (Chen et al., 2017). Therefore, these phenomena suggest that targeting the MAPK pathway and modulating caspase expressions are possible mechanisms of AG in promoting cancer cell apoptosis. Moreover, Xu *et al.* reported that AG reduced the viability of human lung carcinoma cells and promoting apoptosis by inducing DNA damage, increasing the levels ofaspases-3, caspases-9, Bax, Bak, and Cyt-C, and decreasing Bcl-2, Bcl-xL, as well as XIAP. Besides, AG administration downregulated the expressions of p-JAK2, p-STAT1, and p-STAT3, diminished migration and invasion cell number, and upregulated the levels of ROS, which indicated that interfering with JAK/STAT signaling pathway might be a possible mechanism of AG to treat lung cancer (Xu et al., 2021). In brief, these findings revealed that AG accelerated lung cancer cell death by inhibiting the growth and migration of cancer cell as well as inactivating the MAPK, NF-кB, and JAK/STAT signaling pathways.

#### 2.1.5 Liver cancer

Recent studies have illustrated that AG attenuates hepatocellular carcinoma (HCC) cells proliferation by suppressing cell growth and improving apoptosis. AG application arrested the phase of the G1 cell cycle and increased the expression of Sub-G1 DNA. Furthermore, AG promoted oxidative phosphorylation and diminished glycolysis by elevating ROS, MDA, and miR-125b and reducing the expression of HK2, whereas both the inhibitors of ROS and miR-125b and HK2 overexpression abolished the inhibitory effect of AG on HCC cell growth. In addition, AG restrained tumor growth and proliferation *via* modulating the miR-125b/HK2 cascade in HCC xenograft tumor nude mice and Kunming mice transplanted with HCC cells (Li, Hao, et al., 2017). Collectively, these results suggest that AG decreases hepatocellular cancer cells proliferation by reducing HK2 expression *via* augmenting miR-125b expression.

#### 2.1.6 Kidney cancer

Furthermore, AG has been demonstrated to possess an inhibitory effect against kidney cancer, which suppressed the growth of A498 cells by facilitating cell apoptosis and inhibiting the phase of the G2/M cell cycle. AG administration also upregulated the expression of caspase-3, caspase-9, and Bax and decreased Bcl-2, whereas AG did not affect the normal kidney cell growth. Moreover, AG restrained the generation of kidney cancer cell by up-regulating the expressions of microRNA-124, microRNA-203, microRNA-34a, and microRNA-145 (Zhu et al., 2019). Taken together, these results indicate that the protective effect of AG on kidney cancer might be ascribed to its capacity to modulate apoptosis, cell cycle, and microRNA expression.

### 2.2 Effect on obesity, type 2 diabetes mellitus, and diabetic complications

Excessive adipogenesis or lipid accumulation may cause obesity, which further elevates the risks of insulin resistance and DM (Schetz et al., 2019; Chen et al., 2023). More importantly, high fasting glucose levels are one of the diagnoses of DM and may lead to complex complications (Cole and Florez, 2020; He et al., 2021), such as spermatogenic dysfunction (Maresch et al., 2018) and diabetic retinopathy (DR) (Hammes, 2018). Recent studies have demonstrated that AG has great abilities for adipogenesis, obesity, diabetes, and diabetic complications. Swamy *et al.* observed that AG exhibited great potential to prevent obesity, which could inhibit adipogenesis and promote lipolysis in 3T3-L1 adipocytes. AG treatment suppressed lipid accumulation, downregulated the levels of PPAR-γ, C/EBP-α, FAS, and leptin, and augmented the release of free glycerol and the level of adiponectin (Muni Swamy et al., 2022). Moreover, AG attenuated the formation of adipocyte *via* inhibiting the lipid accumulation in the 3T3-L1 adipocyte (Barge et al., 2021). Concretely, the preventive effect of AG against obesity might be conferred by restraining triglyceride accumulation and decreasing the expression of PPARG, C/EBP-α, and FKBP51. Additionally, AG intervention was illustrated to alleviate HG-triggered DM in rats by decreasing the level of blood glucose and increasing the secretion of insulin. Interestingly, the inhibitors of the K^+^ ATP channel, L-type voltage-dependent calcium channels (L-VDCC), PKC, PKA, and the sarcoendoplasmic reticulum calcium transport ATPase (SERCA) reversed the effect of Ca^2+^ influx in pancreatic islets induced by AG, indicating that AG promotes the influx of Ca^2+^ by activating the K^+^ ATP channel, L-VDCC, the protein kinase of PKA and PKC, and SERCA (Rey et al., 2019). Therefore, these results prove that AG boosts the secretion of insulin by enhancing the influx of Ca^2+^.

Furthermore, AG is effective in treating reproductive disease triggered by DM, which has been shown to suppress spermatogenesis dysfunction in STZ-induced diabetic rats by mitigating the decline of body and testis weight and ameliorating reproductive organ injury and sperm damage. In addition, AG treatment reversed the change of seminiferous tubules, basal membrane, and spermatogenic cells and promoted the number, motility, viability, and daily production of sperm, reduced the level of blood glucose and HbA1c, and inhibited oxidative stress and inflammation *via* down-regulating the expression of MDA, NO, TNF-α, and iNO, while up-regulating SOD, GSH-Px, and CAT(Han et al., 2019). Likewise, another study found that AG suppressed the spermatogenesis dysfunction in cyclophosphamide (CTX)-induced diabetic rats by recovering the reproductive organs and improving the quality of sperm. Indeed, AG administration increased the reproductive organs index and the weight of body, testis, and epididymis and elevated the count, motility, and viability of sperm *via* down-regulating the expression of BAD, BCL2-XL, CASPASE9, and CASPASE3 and up-regulating AKT1, BCL-2, ETV1, MAPKAPK2, and RPS6KA5. AG also ameliorated spermatogenesis dysfunction by augmenting the level of testosterone *via* increasing the expression of STAR, PRKACB, and CYP11A1 (Fan et al., 2022). Thus, this range of evidence suggests that AG is a promising therapeutic agent for diabetic spermatogenesis dysfunction. Remarkably, recent research has proved that AG might serve as a therapeutic agent for DR. AG intervention could alleviate the overexpression of VEGF in Müller cells caused by HG, thereby inhibiting abnormal angiogenesis in retina and contributing to the alleviation of DR (Ke et al., 2012).

### 2.3 Effect on neuropathy

Ischemia/reperfusion (I/R) injury is a serious pathological factor for cerebrovascular and heart diseases, which can activate the expression of genes related to apoptosis, produce free radicals, induce inflammation (Wang et al., 2021), and trigger intracellular calcium overload (Shen et al., 2019). Recently, several studies have illustrated that AG may serve as a therapeutic agent for cerebrovascular, spinal cord, and myocardial I/R. AG intervention was shown to mitigate brain I/R injury in rats and dysfunctions in thapsigargin-treated SH-SY5Y cells by inhibiting endoplasmic reticulum (ER) stress and ER-triggered apoptosis, as evidenced by the reduced expression of c-caspase-3, Bax, caspase-12, GRP78, and CHOP and the augmentation of Bcl-2. Additionally, AG improved the long-term neurological condition and suppressed inflammation in brain I/R injury rats by decreasing IL-1β, IL-6, IL-8, TNF-α, COX-2, and NOS (Liu et al., 2020). Furthermore, AG alleviated spinal cord ischemia/reperfusion (SCI/R) injury in mice by ameliorating motor neuron loss and injury and improving the Basso mouse score. Simultaneously, AG suppressed oxidative stress, inflammation, and necroptosis *via* down-regulating MDA, TNF-α, and IL-6, up-regulating SOD and GSH, and reducing the expression of HMGB1, RIP1, RIP3, and MLKL. Interestingly, the inhibitor of RIP1 (necrostatin-1) could mimic these anti-oxidative and anti-necrotic effects of AG in SCI/R mice, indicating that AG might prevent SCI/R injury *via* targeting RIP1-meidated necroptosis (Sun et al., 2021). AG was also demonstrated to alleviate myocardial I/R injury in isolated rat heart by improving cardiac function, ameliorating the myocardial structure, and reducing the infarct size and cardiac injury. AG application increased left ventricular developed pressure, the maximum up/down rate of left ventricular pressure, heart rate, and coronary flow and decreased the levels of LDH and CK. Meanwhile, AG suppressed the oxidative stress, inflammation, and apoptosis *via* up-regulating SOD, GSH/GSSG ratio, and Bcl-2, down-regulating the number of apoptotic cells, and reducing the level of MDA, ROS, TNF-α, IL-6, and Bax (Qu et al., 2016). Altogether, these findings show that AG alleviates I/R injury *via* reducing apoptotic protein expressions, suppressing oxidative stress, and relieving inflammation.

Neuroinflammation is the immune function that protects the brain from injury (Chen et al., 2022), but excessive inflammation may trigger the activation of astrocytes and microglial cells that can worse brain injury or cause neurodegenerative diseases (Calsolaro and Edison, 2016), such as depression and Alzheimers disease (AD) (Leng and Edison, 2021; Wang et al., 2022). Recent studies have elucidated that AG ameliorates AD and depression *via* attenuating neuroinflammation, improving cognitive function, and reducing the deposition of amyloid plaques and neurofibrillary tangles (NFTs). Kim *et al.* reported that AG application relieved neuroinflammation in LPS-treated mice and microglia by reducing the levels of NO, iNOS, TNF-α, IL-1β, IL-6, and COX-2, down-regulating the level of ROS, up-regulating HO-1, and improving the activities of DPPH radical scavenging. Moreover, AG decreased the expressions of p-p38, p-Erk, p-JNK, and p-NF-κB, suggesting that suppressing the HO-1/MAPK and P13K/Akt signaling pathways might be an approach of AG for inhibiting neuroinflammation during AD (Kim et al., 2022). Moreover, considerable studies have illustrated that senile plaques and neurofibrillary tangles (NFTs) play pivotal roles in AD, contributing to the cognitive impairment and neuronal damage. AG could recover the cognitive deficits and damage in SAMP8 mice and Aβ_1−42_O-induced neuronal cells by augmenting Erα and Erβ, decreasing degenerating neurons, and suppressing the senile plaques and NFTs. Mechanically, AG treatment downregulated the expressions of Aβ_1−40_, Aβ_1−4_, β-CTF, Thr231, Ser396, and Ser202/Thr205 and upregulated GSK3β. Additionally, AG also enhanced the abilities of memory and learning, as evidenced by the reduction of escape latency in Morris water maze tests and the increase of the number of mice in target crossings in a probe trial. Interestingly, the inhibitor of estrogen receptor abolished the protective effects of AG (Liu et al., 2022), which indicated that modulating the expression of estrogen receptor may be a way of AG treating the cognitive deficits in AD.

NLRP3 inflammasome underlies numerous debilitating disorders including depression, which elevates the expression of p65, NLRP3, ASC, cleaved IL-1β, cleaved gasdermin D, and caspase-1 and downregulates SIRT1. Tong *et al.* found that AG alleviated the depression in LPS- and ATP-treated BV2 cells, and chronic unpredictable mild stress (CUMS) challenged mice by suppressing the activation of NLRP3 inflammasome, as evidenced by the reduction of NF-κB p65, NLRP3, cleaved IL-1β, cleaved gasdermin D, and caspase-1 and the increase of SIRT1. Meanwhile, AG intervention inactivated the microglia by decreasing the expression of Iba1. Intriguingly, SIRT1 inhibitor countervailed the inhibitory effect of AG on NLRP3 inflammasome, which indicated that targeting the SIRT1-mediated NLRP3 inflammasome axis might be a possible mechanism of AG to treat depression (Tong et al., 2020). AlCl_3_/D-galactose was demonstrated to induce cognitive dysfunction in mice by stimulating neuroinflammation *via* activating the astrocytes and microglia, whereas AG could reverse neuronal damage triggered by toxins. Specifically, AG alleviated the infiltration of inflammatory cells in hepatocytes and small intestinal tissues and ameliorated senescence by improving learning and memory abilities and decreasing the level of β-GAL. Moreover, AG downregulated the levels of MDA, TNF-α, IL-1β, IL-6, Notch1, HES-1, RBP-Jκ, and NF-κB, upregulated T-SOD, T-AOC, CAT, GSH-Px, IL-10, and APP, and reduced the expressions of GFAP^+^ and IBA1^+^ signals, indicating that suppressing the Notch/HES-1-NF-κB signaling pathway and attenuating microglia activation might account for the therapeutic effect of AG in cognitive impairment (Hu et al., 2022). Altogether, these findings reveal that modulating the HO-1/MAPK, P13K/Akt, SIRT1- NLRP3, and Notch/HES-1-NF-κB signaling pathways might be the mechanism of AG in mitigating neuroinflammation, cognitive impairment, depression, and AD.

### 2.4 Effect on respiratory diseases

In recent years, an increasing number of studies have proved that AG exhibits a promising therapeutic effect on multiple respiratory diseases, such as asthma, chronic obstructive pulmonary disease (COPD), and acute lung injury (ALI), and the underlying mechanisms of AG in treating these disorders have been elucidated to a certain extent, which greatly promotes the possibility of AG as a novel agent for respiratory diseases. Asthma, characterized by swelling and elevated mucus in the airways and bronchospasm, is a chronic disorder that severely affects the breathing of patients (Sockrider and Fussner, 2020; Stern et al., 2020). AG was shown to ameliorate OVA-triggered asthma in mice, restraining the process of Th1 and Th2 differentiation by down-regulating the levels of IL-4, IL-5, IL-13, IgE, and SOCS-3 and up-regulating IFN-γ and SOCS-5. Additionally, AG reduced airway resistance and altered the composition of bronchoalveolar lavage fluid by suppressing the increase of leukocytes, eosinophil, and lymphocyte and alleviating eosinophilia in lung tissue (Liu et al., 2015). In addition, another study reported that AG attenuated LPS-induced oxidative stress in the BEAS-2B-cell by modulating the TLR4-PKCβ2-NADPH and MAPK signaling pathways, as reflected by the reduction of ROS, eotaxin-1, PLCγ1, p-PKCβ2, p22^phox^, p47^phox^, and TLR4. Remarkably, these effects of AG on the BEAS-2B-cell were imitated by the TLR4 inhibitor, which indicated that targeting the TLR4-PKCβ2-NADPH axis might be the pivotal mechanism of AG in ameliorating oxidative stress during asthma. Additionally, AG suppressed apoptosis by down-regulating the levels of p-p38, p-JNK, and caspase-3 and up-regulating p-ERK, and p-Akt (Cho et al., 2014). Furthermore, AG was reported to alleviate ovalbumin (OVA)-induced airway fibrosis in mice and the H_2_O_2_-treated BEAS-2B-cell, which attenuated fibrosis by inhibiting epithelial-to-mesenchymal transition (EMT) and ameliorated oxidate stress *via* down-regulating the expression of vimentin and ROS and up-regulating E-cadherin, as well as reducing the deposition of collagen. Furthermore, AG also restrained autophagy by decreasing the level of beclin-1, LC3A/B, and α-SMA (Cho et al., 2015). Therefore, these findings illustrate that modulating SOCS-mediated inflammation, the TLR4-PKCβ2-NADPH and MAPK signaling pathways, and autophagy might be the mechanisms of AG for asthma therapy.

COPD is an inflammatory airway disorder, characterized by lower airway stenosis and excessive mucus (Ferrera et al., 2021). AG has been reported to ameliorate OVA-triggered COPD in mice and H_2_O_2_-induced inflammation in BEAS-2B cells by alleviating airway thickening and suppressing inflammation, as evidenced by the downregulation of ICAM-1, integrins, MCP-1, and α-SMA. In addition, AG prevented pulmonary dysfunction in mice by attenuating the macrophages, neutrophils, and mast cells infiltration by decreasing the levels of F4/80, CD68, and CD11b (Kim et al., 2017). Another study demonstrated that AG alleviated cigarette smoke-induced COPD in mice and thrombin-caused inflammation in A549 cells by suppressing thrombosis formation, inflammation, and oxidative stress. AG administration attenuated the airway thickening, alveolar emphysema, pulmonary embolism and infarction, reduced the expressions of uPA, TF, COX-2, iNOS, and ROS, and increased the level of tPA. Furthermore, AG inactivated the PAR and MAPK signaling pathways by down-regulating the levels of PAR-1, PAR-2, p-p38, p-c-Jun, p-JNK, and p-ERK (Kim et al., 2021). Thus, these findings suggest that AG alleviates COPD by suppressing the PAR and MAPK signaling pathways, and AG might serve as a potential agent for COPD.

ALI is a respiratory disease with significant mortality that is associated with acute and sever inflammation (He et al., 2021). AG could ameliorate ALI triggered by lipopolysaccharide (LPS) in rats by suppressing oxidative stress and inflammation, which upregulate the expression of Nrf2 and HO-1 and downregulate MPO, TNF-α, and MMP-9. Moreover, AG has been shown to inhibit inflammation infiltration and decrease the level of lipid hydroperoxide. Interestingly, the effect of AG in augmenting the level of HO-1 was abolished in Nrf2 knocked-down BEAS-2B cells (Zheng et al., 2019), suggesting that the protective activity of AG against ALI might be partially ascribed to its capacity to regulate Nrf2/HO-1 signaling pathway-mediated inflammation. In addition, another study reported that AG could alleviate LPS-induced ALI in mice by suppressing the NF-κB signaling pathway. AG treatment decreased the lung wet-to-dry weight ratio, the level of BALF total protein, and the numbers of neutrophils and macrophages, downregulated the levels of TNF-α, IL-1β, and IL-6, and repressed the degradation of IκB (Soromou et al., 2012). Therefore, these findings indicate that targeting the Nrf2/HO-1 signaling pathway may be the mechanism of AG for ALI.

### 2.5 Effect on osteoporosis

Osteoporosis (OP), characterized by skeletal fragility and microarchitectural deterioration, is a metabolic bone disease mainly occurring in postmenopausal women (Black and Rosen, 2016). Recent studies have found that AG attenuates OP in ovariectomized mice and bone marrow mononuclear cells (BMMCs) by increasing the level of bone mineral density, relative bone volume, and trabecular number, improving the structure of bone, and alleviating the bone resorption. AG treatment upregulated osteoprotegerin, downregulated TRACP-5b, RANKL, ACP5, F2R, NFATC1, CTSK, c-fos, and c-Src kinase, and suppressed F-actin rings formation in BMMCs (Yang et al., 2022). In addition, AG was reported to attenuate OP in ovariectomized mice and MC3T3-E1 cells by improving osteoblastic differentiation and bone formation, which upregulated Alp, Ocn, Opn, BMP-2, p-Smad1/5/9, Runx2, Erk1/2, p-Erk1/2, p38, p-p38, and JNK in MC3T3-E1 cells, indicating that the BMP and MAPK signaling pathways are involved in the process of osteoblastic differentiation (Liu et al., 2019). Collectively, these findings prove that targeting the BMP and MAPK signaling pathways might be a mechanism of AG for OP, and AG may function as a promising therapeutic agent for bone diseases.

### 2.6 Effect on reproductive system diseases

AG has been proved to possess a therapeutic effect on disorders associated with the reproductive system, especially varicocele (VC), Leptospira-infected uterine, and endometritis. Karna *et al.* found that AG plays a predominant role in treating VC in rats, improving the histology of the reproductive organs, the sperm count, motility, and viability *via* ameliorating vacuolization and the decrease of spermatogenic cells and sperm in seminiferous tubules, augmenting the Johnsens score, and mitigating apoptosis. Moreover, AG suppressed VC-related oxidative stress, ER stress, inflammation, and mitochondrial pathway-mediated apoptosis by up-regulating the expression of MDA, ROS/RNS, TNF-α, IL-6, Grp 78, p-IRE1, P-JNK, caspase-3, and Bax/Bcl2, increased the level of testosterone by elevating StAR, and decreased the follicle-stimulating hormone (FSH) and luteinizing hormone (LH) (Karna et al., 2019). In addition, AG was proved to be a promising agent for uterine infection. Hu *et al.* reported that AG could prevent inflammation in sheep endometrial epithelium cells (SEECs) induced by *E. coli* by inactivating the TLR4/NF-κB signaling pathway. AG administration reversed the decrease of SEECs viability, downregulated the inflammatory cytokines IL-1β, IL-6, IL-8, IL-12α, and TNF-α, and reduced the levels of KKβ, NF-κB, TLR4, and p-IκBα (Hu et al., 2020). Moreover, AG has been shown to possess therapeutic effect on endometritis, alleviating LPS-induced inflammation in uterine epithelium cells and Leptospira-induced uterine inflammation in mice by repressing the NF-κB and MAPK signaling pathways. Specifically, AG downregulated the mRNA and proteins expressions of TNF-α, IL-1β, and IL-6 in mice and uterine epithelium cells and suppressed the infiltration of inflammatory cells and histopathological changes. Moreover, AG reduced the expression of p-IκB, p-p65, p38, ERK, and JNK (Zhang et al., 2017). Altogether, these results reveal that inhibiting the NF-κB and MAPK signaling pathways might be the mechanism of AG for endometritis therapy.

### 2.7 Effect on inflammatory diseases

Osteoarthritis (OA) is a chronic and degenerative joint disease with a high disability rate that dramatically affects the quality of life of patients (Mero et al., 2014). AG has been shown to possess a therapeutic effect on IL-1β-triggered OA in human chondrocyte by attenuating inflammation, in doing so down-regulating NO, PGE2, iNOS, COX-2, p65, β-actin, ERK1/2, JNK, and p38 and increasing the expression of PPAR-γ. Interestingly, the inhibitor of PPAR-γ reversed the inactive effect of AG (Ma et al., 2015), suggesting that targeting the PPAR-γ-mediated p38 and p65 axis might be a possible mechanism of AG in treating OA. Ulcerative colitis (UC) is a type of inflammatory bowel disease, starting with mucosal inflammation in the rectum and extending proximally in the colon in a continuous manner (Kobayashi et al., 2020). Peng et al. reported that AG could alleviate colitis in dextran sulfate sodium (DSS)-induced colitis mice by ameliorating the structure of the colon and intestinal barrier damage, regulating the gut microbiota, and suppressing the metabolic endotoxemia. Indeed, AG application relieved inflammation in the colon by down-regulating MCP-1, TNF-α, IL-1β, IL-6, IFN-γ, COX-2, MPO, LPS, and TLR4 and up-regulating ZO-1, Occludin, and Muc2. Moreover, AG decreased the expression of p-IκBα, p-IKKα/β, and p-p65, indicating that inactivating the NF-κB pathway might be a mechanism of AG to treat UC (Peng et al., 2020). In addition, other research has proved that AG ameliorates colitis in DSS-induced colitis mice and HCT-116 and HT-29 cells by alleviating the shortening of the colon and suppressing inflammation by down-regulating the expression of TNF-α, IL-6, and IL-8. Additionally, AG also inhibited inflammation by abolishing the activity of DNA binding to NF-κB and down-regulating IκBα (Han et al., 2021). Altogether, these results demonstrate that targeting the NF-κB signaling pathway is a possible strategy of AG for UC therapy. Mastitis, characterized by infection and inflammation associated with the mammary gland, is a worldwide widespread disorder that threatens the health of women who are breastfeeding (Spencer, 2008). AG has been demonstrated to a possess protective effect on LPS-induced mastitis in mice by inhibiting NF-κB-mediated inflammatory response, thus significantly attenuating the increase of MPO and down-regulating the levels of TNF-α, IL-1β, IL-6, NF-κB p65, and IκBα (Li et al., 2013). In addition, AG has been shown to suppress the inflammation triggered by LPS in mouse mammary epithelial cells via modulating the TLR4/NF-κB and MAPK signaling pathways. AG also reduced the levels of inflammatory cytokines and mediators, as evidenced by the decrease of NO, TNF-α, IL-6, iNOS, and COX-2. In addition, AG decreased the expression of TLR4, p-NF-κB P65, IκBα degradation, p-ERK, p-P38, and p-JNK (Li et al., 2014). Thus, these findings suggest that inhibiting the TLR4, NF-κB, and MAPK signaling pathways might be a mechanism of AG in inflammatory diseases, and thus, AG may be a useful candidate for inflammatory diseases.

### 2.8 Other effects

Coagulation is a critical process in wound healing that is associated with the balance of anticoagulant and procoagulant (Shah et al., 2014). The level and abilities of platelets are significant to hemostasis after injury (Agbani and Poole, 2017), and elevating the level of platelets is one of the mechanisms of AG in hemostasis. AG has been demonstrated to possess a procoagulant effect *in vitro* and in heparin sodium-induced rats, which shortened the activated partial thromboplastin time and thrombin time and decreased fibrinogen in both instances. Furthermore, heparin sodium triggered the increase of coagulation time and the decrease of plasma viscosity and whole blood viscosity in rats, whereas AG reversed these parameters. Additionally, AG downregulated the levels of 6-Keto Prostaglandin F_1α_, nitric oxide synthase, and prothrombin time and upregulated platelet, endothelin-1, thromboxane B_2_, erythrocyte sedimentation rate, and packed cell volume (Li et al., 2020). Taken together, these results suggest that AG has favorable procoagulant properties and may serve as a promising coagulant. In addition, AG has been shown to possess the effect of sedation and hypnosis in mice, reducing the latency of sleep and lengthening sleep time. In addition, AG lessened the spontaneous activity of mice compared with a control group and lowered the convulsions and prolonged the latency of convulsions (Li et al., 2017). Furthermore, AG improved neuropathic pain in chronic constriction injury (CCl) rats by inhibiting the P2X4/ERK-mediated pathway. Specifically, AG increased the pain threshold of CCl rats and decreased the level of TNF-R1. Meanwhile, AG inactivated the satellite glial cell by down-regulating the expression of P2X4 and GFAP in dorsal root ganglia *via* abolishing the ERK signaling pathway. Interestingly, the results of the inhibitor of P2X4 were consistent with the AG group, which suggested that modulating P2X4-mediated signaling might be a mechanism of AG for neuropathic pain (Wang et al., 2020). Thus, these findings suggest that AG could exert an analgesic effect *via* the P2X4/ERK signaling pathway. Moreover, AG has been shown to possess an outstanding antibacterial effect *in vitro*. It was reported that AG attenuated the ability of *C. albicans* 475/15 *via* suppressing the yeast-to-hyphal transition and destroying cell membrane integrity. AG treatment decreased the number of hyphal cells and inhibited the ergosterol biosynthesis. Moreover, AG restrained drug efflux by down-regulating the expression of *CDR1* (Ivanov et al., 2020). Thus, these findings imply that AG might serve as a promising antimicrobial agent.

AG also displays therapeutic potential for menopause, rheumatoid arthritis (RA), and atherosclerosis. AG has been shown to ameliorate menopausal symptoms in aged rats by suppressing the apoptosis of ovarian granulosa cells (GCs), up-regulating the levels of 17β-estradiol (E2) and progesterone (P4), and down-regulating FSH and LH. Additionally, AG promoted the proliferation of GCs and decreased the number of cells undergoing apoptosis by augmenting the expression of Bcl-2 in cultured GCs (Wei et al., 2016). Thus, these findings indicate that AG may be a potential agent for menopause. Furthermore, Jia et al. observed that AG attenuated RA in collagen-induced arthritis mice by alleviating swollen joints, hind paw thickness, joint space widening, synovial vascularity, bone destruction, and inflammation *via* reducing the levels of TNF-α, IL-1β, IL-6, IL-8, MMP-1, MMP-3, and MMP-13. In addition, AG suppressed the increase of MMPs and the activation of the MAPK and AP-1 pathways triggered by TNF-α in MH7A cells, as reflected by the downregulation of p-JNK, p-p38, and p-c-Jun (Jia et al., 2019). Thus, these results demonstrate that AG relieves RA by reducing the expression of MMPs *via* inactivating the JNK/p38/AP-1 pathways. Furthermore, AG has been demonstrated to attenuate atherosclerosis in apoE^−/−^ mice and THP-1 monocytes and 293T cells by enhancing cholesterol efflux and suppressing the accumulation of lipid and inflammation, which upregulated the expression of ABCA1 and ABCG1 and downregulated IL-6, MCP-1, TNF-α and IL-1β. Moreover, AG alleviated atherosclerotic plaques, augmented HDL-c, and reduced the level of total cholesterol, LDL-c, and triglycerides in apoE^−/−^ mice. Additionally, AG increased PPARγ and LXRα, whereas the increase of ABCA1 and ABCG1 were abolished by the inhibitors of PPARγ or LXRα. AG also decreased the levels of p65 and TLR4, while the inhibitor of NF-κB restrained the anti-inflammatory effect of AG. Notably, the siRNA of ABCA1 and ABCG1 reversed the promotive effect of AG on cholesterol efflux and the inhibition of the surface expression of TLR4 (Zhao et al., 2021). Collectively, these results suggest that AG ameliorates atherosclerosis via inhibiting inflammation and improving cholesterol efflux by inhibiting the TLR4/NF-κB pathway by up-regulating ABCA1 and ABCG1.

In addition, UVB plays an important role in the formation actinic keratosis (AK), which is a symbol of precancerous skin lesion. Recently, Li et al. (2017) observed that AG could suppress AK in HaCaT cells and UVB-induced mice. AG treatment downregulated the expression of p-p38 and decreased the levels of p-MSK1, γ-H2AX, and p-H2AX in UVB-induced HaCaT cells. Remarkably, such phenomena were mimicked by p38 knockdown treatment and the p38 inhibitor, which indicated that AG exerted a protective effect on UVB-induced HaCaT cells by directly binding to the p38 MAPK protein and down-regulating the levels of p-p38. Moreover, AG alleviated the thickening of the epidermis and immunocytes infiltration and reduced the levels of TNF-α triggered by UVB in mice (Li et al., 2018).

## 3 Pharmacokinetics of Astragalin

An increasing number of studies have been conducted on the pharmacokinetics of AG *in vivo* in recent years, and the pharmacokinetic parameters of the relevant studies are shown in Table 3. Tao et al. reported that the time to the maximum concentration (T_max_) of AG in the Fu-Zhu-Jiang-Tang total fraction in rats was 0.39 ± 0.14h, and the elimination half time (T_1/2_) of AG was 0.31 ± 0.04 h (Tao et al., 2017); He et al. reported that the T_max_ of AG in total flavonoids from mulberry leaves was 0.19 ± 0.06h, and the T_1/2_ of AG was 0.87 ± 0.52 h (He et al., 2013); and Xu et al. observed that AG was absorbed from the gastrointestinal tract of the rat and was in the plasma for 5 min after it was detected(Xu et al., 2018), indicating that AG was absorbed and eliminated faster in the rat. Compared with the normal group, the myocardial ischemia–reperfusion injury (MIRI) rats exhibited higher T_max_, C_max_, AUC_(0-t)_, AUC _(0-∞)_, MRT_(0-t)_, and MRT _(0-∞)_ and lower CL (Zheng et al., 2020). However, the renal insufficiency rats had lower values of AUC_(0-t)_, AUC _(0-∞)_, and C_max_ and higher T_1/2_ and T_max_ (Zhang et al., 2020). These phenomena indicate that different pathological states may affect the metabolism of AG *in vivo*. Meanwhile, the total urine and bile excretion rates of AG in a MIRI group were shown to be lower than those in a normal group, whereas the fecal excretion rate was higher than normal group (Lu et al., 2021), suggesting that urine and feces pathways may be the main excretion ways of AG in MIRI rats. Interestingly, different forms of processing Cuscutae Semen have resulted in differing absorption and bioavailability of AG. The T_max_ of AG in stir-frying Cuscutae Semen (SF-CS) was longer than that in salt-processed Cuscutae Semen (SP-CS), while the C_max_ of AG in SF-CS was reduced (Liu, Zou, et al., 2019). These findings illustrate that salt-processing enhances the absorption and bioavailability of AG *via* increasing the solubility of AG in rats.

**TABLE 3:** The pharmacokinetic parameters of Astragalin.

				Pharmacokinetic parameters								
Route of administration	Model	Medicine	Dose	T_max_ h)	C_max_ (μg/mL)	AUC_(0-t)_ (h·μg/L)	AUC _(0-∞)_ (h·μg/L)	CL (L/h/kg)	V (L/kg)	MRT_(0-t)_ h)	MRT _(0-∞)_ h	Reference
Oral	Sham-operated Rat	*Polygonum orientale inflorescence* extracts	3 g/kg	0.58 ± 0.20	107.20 ± 11.27	1056.94 ± 487.52	1178.31 ± 543.50	160.61 ± 68.39	2930.25 ± 688.39	18.00 ± 10.91	20.07 ± 12.16	Zheng et al. (2020)
Oral	MIRI Rat			0.79 ± 0.33	114.29 ± 14.56	1602.4 ± 376.26	1827.14 ± 429.03	143.07 ± 19.10	2810.96 ± 435.27	20.94 ± 7.64	23.88 ± 8.71	
Oral	Rat	*Drynariae rhizoma* extract	4 g/kg	0.67	1203.63 ± 90.89 (ng/kg)	1999.53 ± 338.21	2012.24 ± 331.76	-	-	1.97 ± 0.25	-	Xu et al. (2018)
Oral	Rat	Fu-Zhu-Jiang-Tang total fraction	2.7 g/kg (equivalent to 5.48 mg/kg of astragalin)	0.39 ± 0.14	14.50 ± 1.78 (ng/mL)	16.52 ± 0.39 (ng·h/L)	17.93 ± 0.96 (ng·h/L)	-	-	4.18 ± 0.21	-	Tao et al. (2017)
Oral	Rat	Total flavonoids from mulberry leaves	1.0 g/kg	0.19 ± 0.06	73.28 ± 40.60 (ng/mL)	61.66 ± 31.17 (ng·h/L)	69.87 ± 33.68 (ng·h/L)	-	-	-	-	He et al. (2013)
Oral (SF-CS)	Rat	SF-CS extracts	1.9 g/kg	0.99	0.65 ± 0.39 (ng/mL)	1.27 ± 0.76 (ng·h/mL)	11.2 ± 10.8 (ng·h/mL)	2.30 ± 0.98	-	-	-	Liu et al. (2019)
Oral (SP-CS)	Rat	SP-CS extracts	2.2 g/kg	0.17	1.43 ± 0.21 (ng/mL)	1.31 ± 0.27 (ng·h/mL)	2.33 ± 0.72 (ng·h/mL)	2.13 ± 0.20	-	-	-	

Abbreviations: AUC _(0-∞)_, the area under the plasma concentration–time curve from zero to infinity; AUC_(0-t)_, the area under the plasma concentration–time curve from zero to the time of the final measurable sample; CL, clearance; C_max_, maximum plasma concentration; MRT, average residence time; T_max_, time to reach this concentration; V, apparent volume of distribution; SF-CS, stir-frying Cuscutae Semen; SP-CS, salt-processed Cuscutae Semen.

## 4 Toxicity of astragalin

The acute toxicity of AG in polyherbal mixture has been tested by oral administration with 10 g/kg,20 g/kg, 40 g/kg, and 80 g/kg of polyherbal mixture in rats. The results demonstrated that only one in five rats in the 8og/kg group exhibited mild diarrhea after the first 24 h of administration, while toxicity phenomena were not observed in the other groups. In addition, the chronic toxicity results of AG in the polyherbal mixture test indicated that no obvious change occurred in the pancreas, kidney, and liver histology in the treated rats, but bodyweight was significantly reduced and blood glucose levels slightly decreased, accompanied by an increase of total cholesterol and HDL levels compared with the control group. (Madić et al., 2021). In addition, Kim et al. observed that *cyperus exaltatus* var. *Iwasakii* is rich in AG, but this intervention did not change the vital organ tissues in rats at a 2000 mg/kg dose (Kim et al., 2023). Moreover, according to an *in silico* study, AG has been found to possess multiple pharmacological activities and to be unlikely to exhibit acute toxicity (Ammar 2017). Therefore, these findings indicate that AG may have no toxicity, and further studies are needed to prove the development of AG as a novel agent or functional food.

## 5 Conclusion and perspectives

**FIGURE 3:**
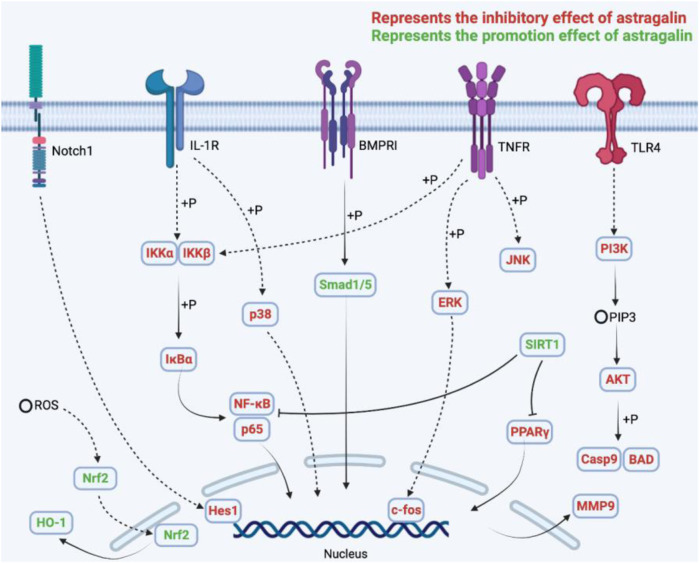
The underlying mechanisms of AG in the treatment of diseases.

Natural products, especially flavonoids, occupy a predominant position in the pharmaceutical and food industries, and they make a substantial contribution to human health. AG is a valuable naturally occurring flavonoid that can be isolated from numerous medicinal plants, flowers, and fruits, such as *Astragalus membranaceus*, *Flaveria bidentis L.) Kuntze*, cherry (*Prunus serotina*), persimmons (*Diospyros kaki*), and *Cuscuta chinensis*. So far, a considerable number of studies have revealed the pharmacological activities and therapeutic potential of AG in diseases therapy, which mainly contains anti-inflammation, anti-oxidative stress, anti-cancers, anti-obesity and DM, anti-I/R injury, and anti-UC properties, as well as preventing neuropathy, respiratory diseases, and mastitis, *etc.* More importantly, the underlying mechanisms of AG in treating/preventing these disorders have been elucidated to some extent, which has greatly facilitated its development as a functional food or lead compound. From these studies, it can be concluded that AG prevents cancers by promoting apoptosis *via* modulating the NF-κB, PI3K/AKT, MAPK, and JAK/STAT signaling pathways. Furthermore, suppressing adipogenesis and lipid accumulation, elevating insulin secretion, and enhancing the influx of Ca^2+^ are considered to be the possible mechanisms of AG in treating obesity and DM. In addition, by inhibiting ER stress, apoptosis, and inflammation and regulating RIP1-mediated necroptosis, AG exerts an outstanding anti-I/R injury effect *in vitro* and *in vivo*. Interestingly, the therapeutic effects of AG on depression and AD might be conferred by modulating the HO-1/MAPK, P13K/Akt, SIRT1-NLRP3, and Notch/HES-1-NF-κB signaling pathways. Moreover, AG could prevent asthma *via* modulating SOCS-mediated inflammation, the TLR4-PKCβ2-NADPH and MAPK signaling pathways, and autophagy (Figure 3).

However, it is noteworthy that AG may show opposite pharmacological activities in some specific situations, such as promoting apoptosis in the treatment of tumors while exhibiting anti-apoptotic effects in the treatment of menopausal symptoms and VC, suggesting that more *in vivo* and *in vitro* studies are needed to elucidate the quantitative-effect relationship of AG. At present, most of the research on the pharmacological activities of AG is focused on *in vitro* experiments, and toxicity studies and safety evaluations of AG are also lacking, indicating that more clinical studies and safety studies are needed to evaluate the potential of AG as a functional food or a novel agent. Additionally, the current research on the pharmacokinetics of AG has illustrated that different pathological states and different processing may affect the metabolism of AG *in vivo*. For example, urine and feces pathways may be the main excretion ways of AG in MIRI rats, suggesting that intestinal flora disorder in the MI condition is one reason affecting AG excretion. Thus, more attention should be paid to the efficacy and safety of AG when using AG in patients with MI. However, the pharmacokinetic studies of AG are still insufficient, indicating that more studies are needed to clarify the metabolism of AG in different disease states or in different pathological states. In brief, AG is a promising multifunctional guardian of human health, and in-depth studies on its pharmacological mechanism of action, toxicity, and dose–effect relationship are beneficial for its application in the food and pharmaceutical industries.

## Figures and Tables

**Table:** 

**4-OH-E2**	4-hydroxy-E2
**AD**	Alzheimers disease
**AG**	Astragalin
**AK**	Actinic Keratosis
**ALI**	acute lung injury
**BMMCs**	bone marrow mononuclear cells
**CCl**	chronic constriction injury
**COPD**	chronic obstructive pulmonary disease
**CTX**	cyclophosphamide
**CUMS**	chronic unpredictable mild stress
**CYP**	Cytochrome P450
**DM**	diabetes mellitus
**DR**	diabetic retinopathy
**DSS**	dextran sulfate sodium
**E2**	17β-estradiol
**EMT**	epithelial-to-mesenchymal transition
**ER**	endoplasmic reticulum
**FSH**	follicle-stimulating hormone
**GCs**	granulosa cells
**HCC**	hepatocellular carcinoma
**I/R**	ischemia/reperfusion
**LH**	luteinizing hormone
**LPS**	lipopolysaccharide
**L-VDCC**	L-type voltage-dependent calcium channels
**MIRI**	myocardial ischemia–reperfusion injury
**NFTs**	neurofibrillary tangles
**OA**	osteoarthritis
**OP**	osteoporosis
**OVA**	ovalbumin
**P4**	progesterone
**RA**	rheumatoid arthritis
**SCI/R**	spinal cord ischemia–reperfusion
**SEECs**	sheep endometrial epithelium cells
**SERCA**	sarcoendoplasmic reticulum calcium transport ATPase
**SF-CS**	stir-frying Cuscutae Semen
**SP-CS**	salt-processed Cuscutae Semen
**UC**	ulcerative colitis
**VC**	varicocele
